# Latissimus Dorsi Tendon Transfer with GraftJacket® Augmentation to Increase Tendon Length for an Irreparable Rotator Cuff Tear

**DOI:** 10.1155/2017/8086065

**Published:** 2017-01-17

**Authors:** John G. Skedros, Tanner R. Henrie

**Affiliations:** ^1^Department of Orthopaedic Surgery, The University of Utah, Salt Lake City, UT, USA; ^2^Utah Orthopaedic Specialists, Salt Lake City, UT, USA; ^3^Intermountain Medical Center, Salt Lake City, UT, USA

## Abstract

Massive irreparable rotator cuff tears can be reconstructed with latissimus dorsi tendon transfers (LDTT). Although uncommon, the natural length of the latissimus dorsi tendon (LDT) could be insufficient for transfer even after adequate soft tissue releases. Descriptions of cases where grafts were needed to lengthen the LDT are therefore rare. We located only two reports of the use of an acellular dermal matrix to increase effective tendon length in tendon transfers about the shoulder: (1) GraftJacket patch for a pectoralis major tendon reconstruction and (2) ArthroFlex® patch for LDTT. Both of these brands of allograft patches are obtained from human cadavers. These products are usually used to cover soft tissue repairs and offer supplemental support rather than for increasing tendon length. Extending the LDTT with GraftJacket to achieve adequate length, to our knowledge, has not been reported in the literature. We report the case of a 50-year-old male who had a massive, irreparable left shoulder rotator cuff tear that was reconstructed with a LDTT. The natural length of his LDT was insufficient for transfer. This unexpected situation was rectified by sewing two patches of GraftJacket to the LDT. The patient had greatly improved shoulder function at two-year follow-up.

## 1. Introduction

**Figure 1:**
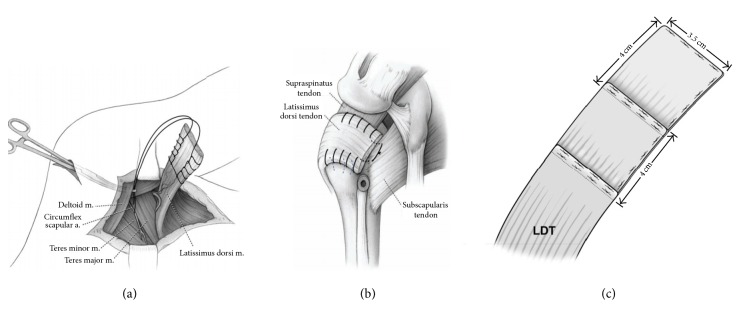
Drawings (a) and (b) show how a LDT is typically transferred and sewn to the defect where the rotator cuff would normally insert. Drawing (c) shows how we extended the tendon with two allograft patches. The running stitches along the margins of the LDT shown in (a) were also done, but this is not shown in (c) (images (a) and (b) are reproduced from [20] with permission of the Journal of Bone and Joint Surgery, Inc.).

Massive irreparable rotator cuff tears can be reconstructed with latissimus dorsi tendon transfers (LDTT) (Figures 1(a) and 1(b)). LDTT for irreparable rotator cuff tears was described nearly three decades ago and has since become a viable treatment option in these cases [1, 2]. LDTT is indicated in younger patients that lack significant glenohumeral arthritis [3]. Sometimes the teres major tendon is transferred in addition to the latissimus dorsi tendon (LDT) in order to obtain better external rotation of the shoulder [4, 5]. Additionally, removal of some humeral bone along with the LDT enables direct transosseous fixation of the LDTT, achieving higher integrity of the transfer [6, 7].

Preoperative active range of motion and sex are important predictors of outcome in LDTT (females have worse outcomes) [8]. Additionally, inadequate subscapularis and deltoid function and fatty infiltration of the teres minor can adversely affect the results of LDTT [8–10]. Additional challenges in LDTT include obtaining an adequate view to release the LDT and achieving sufficient length to reach the eventual attachment point [11, 12]. Adequate tendon length can be reliably achieved by releasing soft tissue attachments along the LDT at its muscle belly [11]. But it is known that the natural length of the LDT, even after adequate soft tissue releases, could be insufficient [11, 12]. A similar problem can occur during pectoralis major tendon reconstruction. In these cases, hamstring and patellar tendon-bone autografts and fascia lata and Achilles tendon allografts have been used to bridge the defect between the damaged muscle and its insertion point on the humerus [13]. Joseph et al. [14] describe the case of a pectoralis tendon rupture that was lengthened with an Achilles tendon allograft to provide additional length in order to repair the defect. The use of acellular dermal matrix allograft patches to extend rotator cuff tears that had insufficient length for repair is well described [15–17].

Descriptions of cases where grafts were needed to extend (i.e., lengthen) the LDT are rare, showing that this situation is very uncommon. For example, we only found one case where an acellular dermal matrix patch was used to increase the natural length of the LDT [12]. The patch used in that case was a 3 mm ArthroFlex patch (a brand of human acellular dermal matrix). This product is usually used to cover soft tissue repairs and offer supplemental support rather than for increasing their length [16, 18, 19].

Extending the LDTT with an alternative common acellular dermal matrix (GraftJacket) to achieve adequate length, to our knowledge, has not been reported in the literature. We report the case of a 50-year-old male who had a massive, irreparable left shoulder rotator cuff tear that was reconstructed with a LDTT. The main novel aspect of our case is that the patient's LDT was found to be inadequate during surgery. This unexpected situation was rectified by sewing two patches of GraftJacket to the free end of his LDT. This yielded greatly improved shoulder function at two-year follow-up.

## 2. Case Report

This left-hand-dominant 50-year-old male (weight: 112 kg; height: 185 cm; BMI: 33 kg/m^2^) fell off of his porch on 31 August 2014 and sustained a massive rotator cuff tear (both the supraspinatus and infraspinatus were torn). The subscapularis and teres minor were deemed to be of good quality. Three months later, he had an attempt at repair, but the surgeon found the tendon tear irreparable. It was likely that the patient had a previous but smaller chronic rotator cuff tear from a sports-related injury many years previously. The patient understood that nothing other than a reverse total shoulder arthroplasty could be done to adequately restore shoulder function.

The patient came to our clinic one month after this unsuccessful attempt at repair. Physical examination at that time showed pseudoparalysis as exhibited by active forward flexion and abduction at 60–65° and superior-posterior shoulder subluxations (Table 1). We recommended a LDTT. If active range of motion was not achieved to his satisfaction but the graft healed, then he would likely achieve a tenodesis effect. This in turn would help reduce subluxations of his glenohumeral joint and thereby reduce pain while likely increasing active motion to a moderate amount [21].

The patient then had an arthroscopic evaluation followed by an open acromioplasty with partial repair of the torn infraspinatus and an open LDTT. The surgery was performed by JGS in accordance with the technique described by Dr. Iannotti and colleagues [8]. A superior approach to the rotator cuff was made by detaching the deltoid origin from the anterior aspect of the acromion and with splitting of the middle deltoid fibers for 3.5 cm. The coracoacromial ligament was released with the deltoid and reattached at the conclusion of the operation. The bursa was excised, and the rotator cuff was inspected. A second incision was made along the lateral border of the latissimus dorsi muscle, extending to the posterior axillary crease. The LDT insertion was identified with the arm abducted and internally rotated, and it was detached sharply from the humerus. The neurovascular pedicle was identified and protected, and the muscle was released from its deep fascia attachments. A number 2 nonabsorbable suture was passed with use of a Krakow suture technique along each side of the tendon from the musculotendinous junction to the end of the tendon (Figure 1(a)). Blunt dissection was performed to construct a tunnel deep to the deltoid and superficial to the posterior rotator cuff musculature.

In the conventional surgical technique, the latissimus dorsi muscle and tendon are routinely brought over the top of the humeral head and repaired anterior to the subscapularis, lateral to the greater tuberosity, and medial to the torn edges of the rotator cuff (Figure 1(b)). However, during surgery, our patient's LDT was found to be only 5 cm long, less than the mean LDT length reported by Goldberg et al. [22] (mean: 7.3 cm; range: 6.6–7.8 cm; SD: 0.38 cm) and nearly 5.5 cm shorter than what was needed for an adequate reconstruction. The patient was consented for use of an acellular dermal matrix graft (GraftJacket) but not for other allograft or autograft tissues. The anticipated use of GraftJacket was for augmentation at the repair site. However, GraftJacket has been mechanically tested and was found to be superior in strength to comparable xenografts and allografts (CuffPatch™, Restore, Permacol™, and TissueMend®) [23]. Additionally, an added layer of thickness from folding the GraftJacket allograft was deemed sufficiently strong to extend the length of the LDT.

Two 4 × 7 cm patches of GraftJacket were used to extend the LDT. The thickness of each patch was 2.0 mm (GraftJacket Maximum Force Extreme; Wright Medical Technologies, Inc.; http://documents.wright.com/Document/Get/010660). Each patch was folded in half along its short dimension, which resulted in 4 × 3.5 cm patches that were 4.0 mm thick each. The folded margins of the patches were sewn together with number 2 nonabsorbable sutures (Figure 1(c)). The “biologically resorbable” surface of the GraftJacket was exposed so that it would be in direct contact to the bone at the insertion site for the tendon reconstruction. This surface represents the anatomically deeper part of the graft and it is placed facing the bone in order to allow incorporation of the graft [24].

The first patch was overlapped on the free end of the LDT by 12.5 mm and was then sutured with two rows of nonabsorbable number 2 sutures (Figure 1(c)). The second GraftJacket patch was overlapped by 12.5 mm to the first patch and was sewn with two rows of nonabsorbable number 2 sutures. The total extension of the LDT was then 5.5 cm. A Krakow suture technique with number 2 suture was then passed along the sides of the latissimus tendon to the free end of the graft extension as shown in Figure 1(a). As described below, the extended LDT was then attached directly to the “footprint” that was created for the supraspinatus, upper infraspinatus, and upper subscapularis (Figure 1(b)).

Before attaching the extended LDT to the footprint, residual infraspinatus tendon at the lower portion of the natural insertion site was mobilized by sharp dissection and moved 15 mm upward and sutured to the upper natural insertion site for this muscle. This was done with number 2 FiberWire sutures through drill holes posteriorly and also with the sutures from a Mitek Healix™ anchor that was placed at the location where the supraspinatus and upper infraspinatus naturally merge. This anchor was double-loaded with number Orthocord® sutures.

The LDT with the GraftJacket extension was then pulled upward beneath the posterior deltoid and anteriorly across the defect in the rotator cuff. Along the lateral portion of the footprint, four pairs of horizontal mattress sutures (number 2 FiberWire) were passed through drill holes. The GraftJacket extension was then fastened in place with these number 2 FiberWire sutures and with the two sutures from the aforementioned Mitek Healix anchor. Additional fixation included a series of separate number 2 FiberWire sutures passing through the upper subscapularis and also a series of separate sutures passing medially through the residual supraspinatus tendon near the glenoid. The shoulder was then passively tested for stability and motion; elimination of the posterior-superior subluxations showed that a tenodesis effect was clearly achieved.

After wound closure, the patient was placed in a rigid shoulder orthosis in neutral rotation and 45° abduction for two months, following Gerber et al.'s [9] postoperative treatment. Gentle passive shoulder motion was allowed only after 6 weeks. Active overhead motion was not allowed until four months after surgery. Weight bearing exercise at the shoulder was allowed at nine months after surgery but no lifting over 20 lbs (9 kg) overhead until one year after surgery.

**Figure 2:**
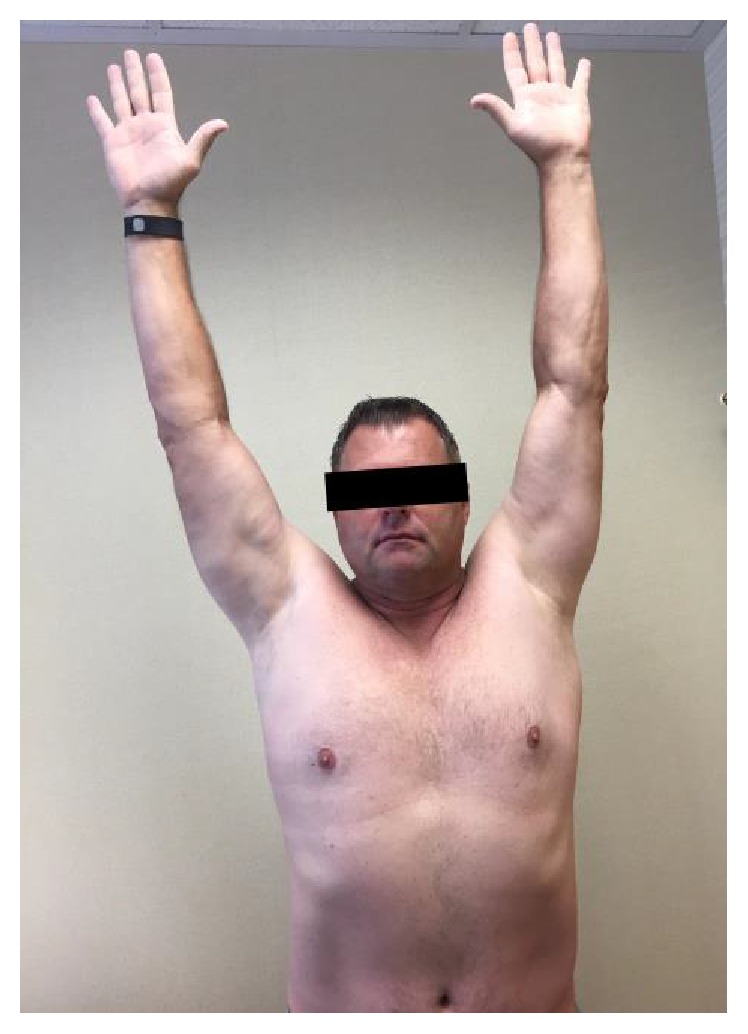
Photograph showing the patient's left shoulder motion at 18-month follow-up (active forward flexion is 165°). Preoperative photographs were not available, but active forward flexion was only 65°.

At follow-up 18 months later, the patient had regained significant strength and range of motion in his left shoulder (Table 1 and Figure 2). He reported being very satisfied with the overall results achieved from his surgery. However, he avoided sleeping on his left side because his shoulder “aches really bad” and he reported shoulder fatigue with repetitive overhead reach. He was not taking regular pain medication besides occasional over-the-counter NSAIDs.

**Figure 3:**
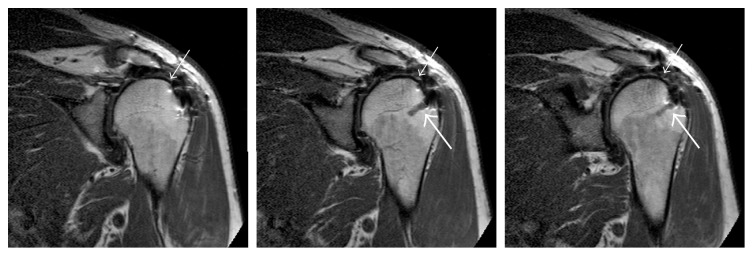
A series of adjacent coronal MR images taken at 18 months after surgery showing the graft (thin white arrow) and suture anchor (thicker white arrow). Mild arthritic changes are also seen.

At 18 months after his LDTT, magnetic resonance (MR) scanning was done solely for the purpose of examining integrity of the graft. The graft was found to be intact and without evidence of thinning (Figure 3). Mild glenohumeral arthritis was noted in the MR images, which likely contributed to some of his continuing low-level pain. At final follow-up at two years after surgery, the pain had improved to only trace and his function was maintained.

## 3. Discussion

At the time of our patient's tendon reconstruction surgery, there was no anticipation that the length of the LDT would be inadequate after releasing it sharply from its insertion and mobilizing the latissimus dorsi muscle belly of the various soft tissue attachments that could restrict the transfer. Although removing some humeral bone along with the LDT would have increased LDT length, it would not have provided the additional 5.5 cm needed for the LDTT. For this reason, we used GraftJacket allograft acellular dermal matrix to extend the LDT. This material has been mechanically tested and was found to have superior resistance in tension loading compared to comparable allografts and xenografts like CuffPatch, Restore, Permacol, and TissueMend [23]. GraftJacket has been used in augmentation of rotator cuff repairs and has been shown to have superior results to nonaugmented repairs [19].

When GraftJacket is used in the context of LDTT for irreparable rotator cuff tear, it would be expected that it would be for augmenting the new attachment site. To our knowledge, the use of GraftJacket to extend the LDT has not been reported. Similar to our case, Petri et al. [12] described lengthening of a LDT using another brand of acellular dermal matrix (ArthroFlex patch) [18].

Additionally, our patient's increased function after the LDTT could be attributed to the latissimus dorsi acting as an active rotator and elevator or because the LDTT simply provided a tenodesis effect that stabilized the shoulder joint. Henseler et al. [25] evaluated eight patients at one year after LDTT. Although they found that the latissimus dorsi has activity after LDTT, they observed a passive tenodesis effect. The tenodesis effect that is achieved resembles that described by Mihata and colleagues [26] as a “superior capsular reconstruction.” One potential problem that could occur in our patient's future is thinning of the graft to the point that the tenodesis effect is lost [21]. Loss of the tenodesis effect of the LDTT can start at approximately six months following the operation, which could also be attributed to elongation of the muscle [21]. There was no evidence that this was occurring in our patient at his 2-year follow-up.

## 4. Conclusion

Our patient had marked improvement in his shoulder range of motion after LDTT with GraftJacket extension of the LDT. Thus, the use of GraftJacket acellular dermal matrix to increase LDT length may be indicated in LDTT cases, where sufficient LDT length is unobtainable. However, studies with much longer follow-up are needed to determine if these good results are maintained.

## Figures and Tables

**(a):** 

Date	Action	Range of motion	Strength
1 month preop^*∗*^	Forward flexion	65	2/5
	Abduction	60	
	External rotation	50	3/5
	Internal rotation	30	
	Extension	35	
	Adduction	35	

18 months postop^†^	Forward flexion	180	4/5
	Abduction	170	
	External rotation	60	4/5
	Internal rotation	70	
	Extension	45	
	Adduction	45	

^*∗*^Preop, preoperatively.

^†^Postop, postoperatively.

**Table:** (b) Shoulder survey scores

	Preop^*∗*^	Postop^†^	
*10 cm VAS score for pain*	6.5	2	
*ASES score*	26.6	68.3	(Best is 100)
*WORC score*		923 (56.1%)	(Best is 0 (100%))
*Simple shoulder test*		10 out of 12	(12 is best)
*DASH score*			(Best is 0; worst is 100)
Total		14.17	
Work module		0	
Sports/performing arts module		37.5	
*Short-Form 36 (SF-36) *			(Best is 100 for all)
Physical functioning		95	
Physical role		100	
Bodily pain		61	
General health		82	
Vitality		60	
Social functioning		75	
Emotional role		100	
Mental health		68	

^*∗*^Preop, preoperatively.

^†^Postop, postoperatively.
